# Estimation of countrywide N_2_O emissions from wastewater treatment in Switzerland using long-term monitoring data

**DOI:** 10.1016/j.wroa.2021.100122

**Published:** 2021-09-30

**Authors:** Wenzel Gruber, Luzia von Känel, Liliane Vogt, Manuel Luck, Lucien Biolley, Kilian Feller, Andrin Moosmann, Nikita Krähenbühl, Marco Kipf, Reto Loosli, Michael Vogel, Eberhard Morgenroth, Daniel Braun, Adriano Joss

**Affiliations:** a Eawag, Swiss Federal Institute for Aquatic Science and Technology, 8600 Duebendorf, Switzerland; b Institute of Environmental Engineering, ETH Zürich, 8093 Zürich, Switzerland

## Abstract

Nitrous oxides (N_2_O) emissions contribute to climate change and stratospheric ozone depletion. Wastewater treatment is an important, yet likely underestimated, source of N_2_O emissions, as recent, long-term monitoring campaigns have demonstrated. However, the available data are insufficient to representatively estimate countrywide emission due to the brevity of most monitoring campaigns. This study showed that the emission estimates can be significantly improved using an advanced approach based on multiple continuous, long-term monitoring campaigns. In monitoring studies on 14 full-scale wastewater treatment plants (WWTPs), we found a strong variability in the yearly emission factors (EFs) (0.1 to 8% of the incoming nitrogen load) which exhibited a good correlation with effluent nitrite. But countrywide data on nitrite effluent concentrations is very limited and unavailable for emission estimation in many countries. Hence, we propose a countrywide emission factor calculated from the weighted EFs of three WWTP categories (carbon removal, EF: 0.1–8%, nitrification only: 1.8%, and full nitrogen removal: 0.9%). However, EF of carbon removal WWTPs are still highly uncertain given the expected variability in performance.

The newly developed approach allows representative, country-specific estimations of the N_2_O emissions from WWTP. Applied to Switzerland, the estimations result in an average EF of 0.9 to 3.6% and total emissions of 410 to 1690 tN_2_O-N/year, which corresponds to 0.3–1.4% of the total greenhouse gas emissions in Switzerland. Our results demonstrate that better data availability and an improved understanding of long-term monitoring campaigns is crucial to improve current emission estimations. Finally, our results confirm several measures to mitigate N_2_O emissions from wastewater treatment; year-round denitrification, limiting nitrite accumulation, and stringent control of sludge age in carbon removal plants.

## Highlights

• Yearlong, continuous N_2_O emission monitoring data from 14 WWTPs.
• High correlation of yearly emission factor with effluent nitrite concentration.
• WWTPs binned in 3 categories according to the nutrient removal goals.
• Method proposed for refined countrywide emission estimation.
• Carbon removal WWTP can emit substantially more N_2_O than expected.

## Graphical abstract

**Figure:**
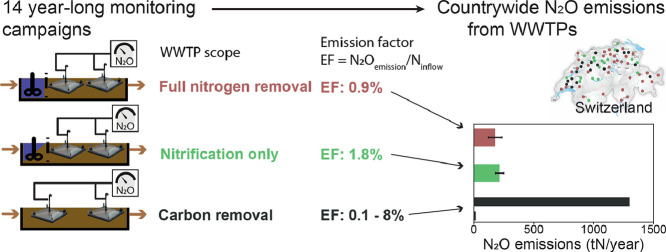

## Introduction

Nitrous oxide (N_2_O) emissions contribute substantially to climate change (IPCC 2014) and stratospheric ozone depletion (Ravishankara et al., 2009). The atmospheric N_2_O concentration is expected to rise until the middle of the 21st century (Tian et al., 2020). Wastewater treatment plants (WWTPs) and their N_2_O production are currently often underestimated emission processes in national greenhouse gas (GHG) inventories. Estimations of countrywide N_2_O emissions are based on assumed emission factors (EFs). EFs in the standard reporting guidelines are much lower (0.03% to 0.14%; Daelman et al., 2015, IPCC 2006) than reported long-term full-scale measurements. With the refinement of the IPCC methodology in 2019, increased EFs of 0.01% to 2.9% appeared in the reporting guidelines (IPCC 2019).

In wastewater treatment, N_2_O formation is primarily caused by biological nitrogen removal through nitrification and denitrification (Kampschreur et al., 2009). The main production processes are the biological stage in the water lines of WWTPs (Kosonen et al., 2016) and the side-stream treatment for reject water from the sludge lines (Joss et al., 2009; Kampschreur et al., 2008b). Emissions of N_2_O produced on WWTPs can occur either on site or in receiving waters if dissolved N_2_O is discharged (Mikola et al., 2014; Marescaux et al., 2018). However, roughly 80% of the N_2_O emissions from WWTPs are released in the aerated zone of biological treatment (Baeten et al., 2020; Chen et al., 2019).

Quantifying representative EFs at the biological stage of WWTPs requires continuous long-term monitoring campaigns due to the substantial daily variation and seasonality of emissions (Daelman et al., 2013). A strong seasonal emission pattern with high emission in spring and low emissions in autumn was previously shown in several long-term monitoring campaigns (Gujer, 2020). However, only six continuous monitoring campaigns of at least one year's duration have been reported to our knowledge (Chen et al., 2019; Daelman et al., 2015, Gruber et al. 2020, Kosonen et al., 2016). Five of these studies were conducted in temperate climates, with a monthly mean temperature of over 10 °C during 4–7 months (Belda et al. 2014). The EF assessed (0.8–2.9% of the total nitrogen loads) were shown to be substantially higher than those found by many short-term campaigns (Chen et al., 2019; Daelman et al., 2015, Gruber et al. 2020, Kosonen et al., 2016, Vasilaki et al., 2019). The causes of the wide range of EFs assessed are still unknown (Vasilaki et al., 2019). Hence, we conclude that additional online long-term monitoring campaigns are much needed to better characterize the variability from N_2_O emissions in full-scale WWTPs. Ultimately, a broad data basis is crucial for countrywide assessment of the N_2_O EFs from wastewater treatment.

Various approaches have been suggested for the extrapolation of N_2_O emissions from WWTPs at countrywide level (Ramirez-Melgarejo et al., 2020). The standard approach is described by the IPCC guidelines (IPCC 2006), which are designed to be calculated with generally accessible variables. Consequently, the approach leads to high uncertainties of the total emission estimates for two reasons. Firstly, in a top-down approach, the nitrogen load to WWTPs is estimated from a country's protein consumption, resulting in substantial differences compared to a bottom-up approach, using measured nitrogen influent loads to WWTPs (Ramirez-Melgarejo et al., 2020). Secondly, the IPCC guidelines suggest a uniform EF independent of the type of WWTP (IPCC 2006). The assumption of a uniform EF leads to high uncertainties, due to the wide range of EFs reported for different types of WWTPs (Cadwallader and Van Briesen 2017) and poorly described key factors characterizing N_2_O EFs (Vasilaki et al., 2019). Several methods have been suggested to overcome these shortcomings arising from short-term monitoring campaigns (Ahn et al., 2010; Valkova et al., 2020). However, the integration of continuous long-term monitoring campaigns on different process types is needed to obtain the most accurate estimates for countrywide N_2_O EFs.

Here, we propose a refined approach to estimating N_2_O emissions from WWTPs based on the EFs of a dozen long-term monitoring campaigns on full-scale WWTPs in Switzerland. To acquire a broad data basis, we conducted seven monitoring campaigns of at least one year's duration on full-scale WWTPs using an adaptation of the flux chamber method (Gruber et al. 2020). Additionally, we extracted the same results from seven long-term monitoring campaigns reported in literature (Daelman et al., 2015; Kosonen et al., 2016, Gruber et al., 2020). We used Spearman's correlation analysis to characterize key variables of a WWTP to predict N_2_O EFs. We use our results to link the variables to patterns detected in our monitoring campaigns to provide experimental evidence for the correlations found, and we conducted full-scale tests on one WWTP to prove concepts from the correlation analysis. Finally, we propose a method for calculating N_2_O emissions for Switzerland and compare it with the methods described in the IPCC guidelines (IPCC 2006, 2019) regarding total emissions and corresponding uncertainties.

## Material & methods

### Monitoring approach

**Fig. 1:**
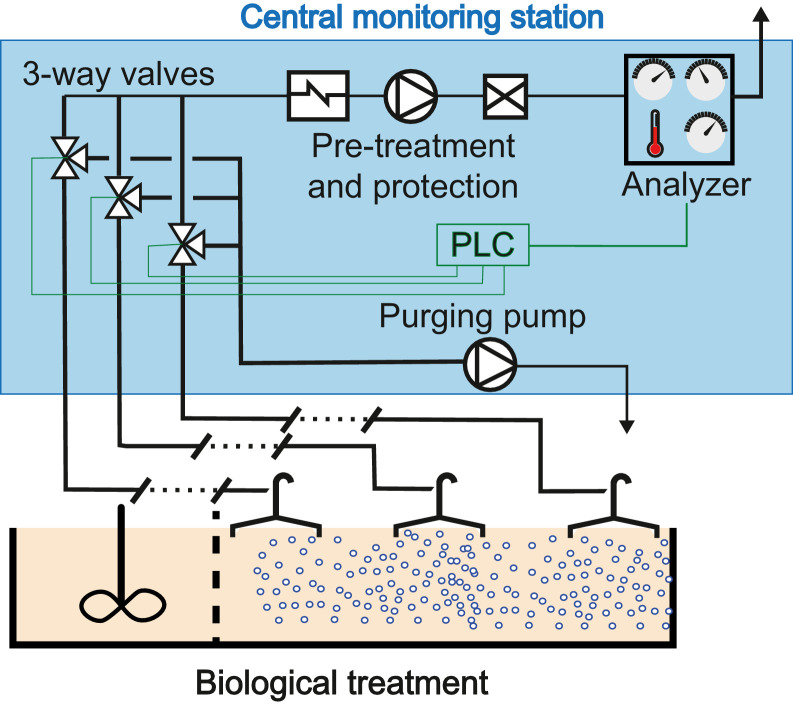
Schematic overview of the monitoring setups applied. Blue, dotted line indicates all elements within central monitoring station. PLC: programmable logical controller.

N_2_O emissions were assessed using an adapted version of the flux-chamber method as described in Gruber et al. 2020 (Fig. 1). In total, five different setups based on the same general concept were applied to monitor the emission on seven WWTPs (see diagrams in the Supporting Information: SI). In short, all discontinuously fed reactors were equipped with a flux chamber. On WWTPs with continuously fed lanes, one or more lanes were monitored, each with three or more flux chambers per lane placed on the aerated compartments.

In contrast to the standard method (Chandran et al., 2016), a chamber (surface: 1 m^2^) with an open outlet was used, and therefore sample gas was not recirculated to the flux chamber after measurement. A sample of the gas flowing through the chamber was diverted at the outlet through a tube with a length of up to a few hundred meters to the central monitoring station, where the off-gas was measured in a non-dispersive infrared analyser (X-stream, Emerson, St. Louis MO, USA). Prior to the measurement, the off-gas was dehumidified by cooling to 4 °C (JCP SL, JCT, Wiener Neustadt, AUT or TC-Standard (PKE 521), Bühler, Ratingen, GER). To monitor the entire biological treatment at multiple sampling points and on various lanes, up to 14 floating hoods can be connected to our system over three-way valves (Parker Lucifer type 7131KBG2JV00, Cleveland, USA). While one of the valves is open to the measurement line, the other channels are purged with a pump (ME 2 NT, Vaccubrand, Wertheim, GER) to ensure a constant flow through all the tubes. The constant flow is considered important for two reasons: (i) to reduce system response time and thus allow fast switching between valves (1 min per measurement) and ii) to avoid freezing of sampling tubes due to humidity in tubing and cold temperatures.

The system is controlled with a programmable logical controller (PLC type WAGO 750–881) which provides two key functions: (i) switching between the valves, and (ii) analogue–digital signal conversion and data storage. Key control variables of the system are a flowmeter (MEMS flow sensor D6F-10A6–000, Omron, Kyoto, JP) in the sample gas duct, a paramagnetic oxygen sensor in the gas analyser (X-stream, Emerson, St. Louis MO, USA), and a humidity sensor (KW1, JCT, Wiener Neustadt, AUT) after the pre-treatment. The flowmeter allows detection of malfunctioning across the whole system or in specific channels when the measured flow drops below a set threshold. The oxygen sensor signals substantial leaks in the channel when oxygen concentration is close to atmospheric concentrations and does not show variation. Finally, the humidity sensor is used as a binary sensor. In case of humidity after the pre-treatment, the measurement is switched off to prevent the analyser malfunctioning. As a second barrier against water entering the analyser, a protection filter is installed directly after the pre-treatment. A field computer is used to parametrize the PLC. Additionally, the field computer establishes data transmission to a data server via the Secure File Transfer Protocol (SFTP) and a 3 G modem (IMON-U300, Insys Icom, Regensburg, GER). On the data server, the monitoring data is processed and synchronized with the operational data of the WWTPs and visualized on demand. The operators of the WWTPs assessed lab data on concentrations of total nitrogen, nitrogen species, and COD sampled from influent, effluent primary clarifier, effluent biological treatment, and effluent WWTP at various intervals. Operational data on influent flow, air supply, wastewater temperature, dissolved oxygen concentrations was acquired over the WWTP's supervisory control and data acquisition (SCADA) system. A detailed description of the monitoring setup can be found in the SI.

### WWTP selection and monitoring campaigns

The seven WWTPs monitored were selected to represent the range of nutrient removal goals set and common types of processes installed in WWTPs in Switzerland (Table 1). Common removal goals are i) carbon removal ii) nitrification and iii) denitrification. Common processes include various types of activated sludge (AS) systems, such as plug-flow (conventional activated sludge (CAS), anoxic-aerobic (AO), anaerobic-anoxic-aerobic (A2O)), alternatingly fed intermittently aerated (A/I) and, sequencing batch reactors (SBR)) and biofilm systems (hybrid fixed bed and activated sludge (IFAS), fixed bed (FB)). We sought to represent various types of WWTP size classes (Table 1). Monitoring campaigns were conducted over at least 1 year. Flux chambers were placed on lanes in accordance with the monitoring strategy proposed in Gruber et al. (2020) (see section 2.1 and SI). The monitoring campaigns reported in Gruber et al. (2020) were included in the selection. Additionally, data from three long-term monitoring campaigns of previous studies was included for the statistical analysis of the data (Chen et al., 2019; Daelman et al., 2015; Kosonen et al., 2016). These studies include data on one AO process and two carrousel (CARR) activated sludge processes. Detailed information on the WWTPs monitored and the results of the newly conducted monitoring campaigns can be found in the SI.

**Table 1:** Characteristics of monitored WWTPs and origin of monitoring campaigns used in this study. Countries: CH, Switzerland; DK, Denmark; NL, the Netherlands; FI, Finland. Processes: A/I, alternatingly fed and intermittently aerated activated sludge treatment; A2O, anaerobic, anoxic and aerobic cascaded activated sludge treatment; AO, anoxic and oxic cascaded activated sludge treatment; CARR, carrousel activated sludge treatment; CAS, conventional activated sludge; FB, fixed bed biofilm reactor; IFAS, integrated fixed film activated sludge; SBR, sequencing batch reactor.

WWTP (country)	Design size in person equivalent's (PE)	Process	Removal goal	Origin
Altenrhein (CH)	80,000	CAS	Nitrification	(Gruber et al. 2020)
Altenrhein (CH)	40,000	FB	Nitrification	(Gruber et al. 2020)
Avedore (DK)	350,000	CARR	Denitrification	(Chen et al., 2019)
Bazenheid (CH)	50,000	IFAS	Denitrification	this study
Birs (CH)	150,000	SBR	Denitrification	this study
Giubiasco (CH)	100,000	CAS	Carbon removal	this study
Hofen (CH)	50,000	AO	Denitrification	this study
Kralingseveer (NL)	360,000	CARR	Denitrification	(Daelman et al., 2015)
Luzern (CH)	280,000	A/I	Denitrification	(Gruber et al. 2020)
Moossee (CH)	50,000	AO	Denitrification	this study
Schönau (CH)	180,000	A2O	Denitrification	this study
Uster (CH)	50,000	SBR	Nitrification	(Gruber et al. 2020)
Vikinmäkki (FI)	840,000	CAS	Denitrification	(Kosonen et al., 2016)
Zurich (CH)	670,000	A/I	Denitrification	this study

### N_2_O emission and EF calculation

The net N_2_O flux for a specific lane was computed with the approach presented by Aboobakar et al. (2013) from the airflow supplied to each reactor section equipped with a flux chamber and the respective N_2_O concentration. When gaps between two consecutive data points did not exceed 30 min, N_2_O concentrations were linearly interpolated; data gaps longer than 30 min were excluded (see Table S1 in the SI). The air flow supplied to the reactor was estimated with three different methods depending on the WWTP: i) from the blower speed and the blower specification provided by the plant operators, ii) by measurement of the total air flow provided to a treatment lane, or iii) by measurement of the air flow provided to a compartment of the treatment lane. The airflow was recorded at intervals of 1 min, and emissions were calculated in 1 min intervals only for the aerated phases. Emissions from the whole treatment plant were extrapolated according to suggestions made in Gruber et al. (2020): when wastewater loadings were expected to differ or operation strategies to change, all lanes were monitored, but lanes with comparable process performance and operation were assumed to emit comparable quantities of N_2_O.

N_2_O EFs were calculated with Eq. (1) for the whole duration of the monitoring campaigns (Gruber et al. 2020; Aboobakar et al., 2013). For the monitoring campaigns that were substantially longer than 1 year (+ 3 months), we evaluated the EFs for all possible data sets of 1 year and calculated their average and standard deviation. In the newly conducted monitoring campaigns of more than 15 months, the yearly average EFs did not exhibit substantial variation. EFs always refer to the yearly average influent nitrogen load of the WWTPs calculated based on 24-hour composite samples. The samples were taken and analysed by the operators of the WWTPs every 5 to 14 days. Where nitrogen influent measurements were not available, we estimated the influent nitrogen load by evaluating a nitrogen mass balance over the primary clarifier based on typical values for mass flow coefficients from standard textbooks (Gujer 2007; Tchobanoglous et al., 2014), because at least effluent loads of the primary clarifier were available in every case (Figure S3, SI). All monitoring data and the values for the nitrogen loads can be found in the SI.

(1) $$EF_{N_{2}O}\;=\frac{\;\sum_{d=1}^{365}\sum_{m=1}^{1440}\left(C_{N_{2}O,m,d}\;\cdot \;Qair_{m,d}\right)}{365\;\cdot \;Nload_{daily}}$$

where EF_N2O_ is the N_2_O EF [kg N_2_O—N/kgN], C_N2O,m,d_ is the measured N_2_O concentration in the off-gas during minute *m* at day *d* [kg N_2_O—N/m^3^], Q_air,m,d_ is airflow supplied by the blower of the aeration system of the WWTP to reactor surface area representative for a sampling point during minute *m* at day *d* [m^3^/d], and Nload_daily_ is the yearly average nitrogen load per day (kgN/d).

### Meta data and correlation analysis

The correlation analysis included key figures and performance indicators that were collected for each WWTP. Spearman rank correlation was used to study the relationships between variables. The following variables on the design and operation of the WWTPs were included: design load, process for biological treatment, type of biomass, biological reactor volume, aerated volume in biological treatment, non-aerated volume in biological treatment, volume of secondary clarifier (if present), nutrient removal goal, aerobic solids retention time (aerobic SRT), total solids retention time (SRT) and information on post, side-stream and sludge treatment (fast sand filtration, supernatant treatment type, co-digestion). The corresponding data for all WWTPs is available in Table S1, SI. If possible, we collected and calculated values for the total nitrogen and COD loads at the influent and the effluent of the biological treatment and of the WWTPs. Additionally, we collected values on nitrite (NO_2_^−^) concentration in the effluent of the biological treatment. The input variables for the correlation analysis are summarized in Table S2, SI. All statistical calculations were performed using Python programming language (version 3.8.3) (Van Rossum and Drake 2009) and the Pandas (McKinney 2010), Numpy (Harris et al., 2020), and Scipy packages (Virtanen et al., 2020).

### Full scale tests at the Hofen WWTP

We conducted full-scale experiments to compare the effects on N_2_O emissions of a pre-denitrification zone with those of a fully oxic process at the Hofen WWTP (see SI for further details). The plant has an AO activated sludge process consisting of six lanes where two lanes use the same clarifier and sludge recycle (SI). Every lane consists of three zones with an equal size, of which the first zone is typically anoxic. Four of the six lanes were monitored: lane 1–1, lane 2–1, lane 2–2, and lane 3–2. Whereas lane 2–1 and lane 2–2 share the same secondary clarifier, lane 1–1 and lane 3–2 are independent of each other. Between February and April 2020, the first of three zones on two lanes (lanes 2–1 and 3–2) were fully aerated. The exact dates and durations for each experiment are given in Table 2.

**Table 2:** Start date, end date, duration, and experimental lanes during experimental phases with one lane fully aerated at the Hofen WWTP.

Start date	End date	Days	Lane fully aerated
4.2.2020	14.4.2020	70	2–1
14.4.2020	27.4.2020	13	3–2
17.6.2020	24.6.2020	8	2–1
9.9.2020	23.9.2020	13	2–1

### Extrapolation to the whole of Switzerland and uncertainty estimation

Countrywide N_2_O emissions were estimated by multiplying the total nitrogen load to the WWTP and different estimates of the EFs from WWTPs for Switzerland. We used four approaches for the estimation of EFs: a) the 2006 IPCC guidelines (IPCC 2006), b) the 2019 refinement of the IPCC guidelines (IPCC 2019), c) the average EF of all monitoring campaigns analysed in this study with a bottom-up approach for activity data estimation, based on extrapolation from data on 70% of the Swiss wastewater load treated in WWTPs (Strähl et al., 2013), and d) a method developed in this study that used the same nitrogen loads as in c). Emissions outside of the biological treatment of a WWTPs were not considered. All equations refer to the standard IPCC terminology.

#### Approach a

The 2006 version of the IPCC guidelines (IPCC 2006), the current standard method, was applied according to Eq. (2) of this study. The values for the estimates were chosen according to the Swiss implementation of the 2006 IPCC guidelines. The EF applied was 0.032%, as suggested by the guidelines. A standard deviation could not be calculated for the resulting countrywide emission, because the 2006 EF is based on a single monitoring campaign. Numerical values for other variables are given in Table 3.

(2) $$N_{2}O_{PLANTS}=Protein\cdot F_{NPR}\cdot \;T_{PLANTS}\cdot F_{IND-COM}\cdot \;EF_{PLANTS}$$

where N_2_O_PLANTS_ is the total, direct N_2_O emissions from WWTPs in Switzerland per year (kgN_2_O—N/year), Protein is Swiss protein consumption (kg protein/year), F_NPR_ is the fraction of nitrogen in protein (-), T_PLANTS_ is the connection rate to WWTPs (-), F_IND-COM_ is the factor for industrial and commercial protein (-), and EF_PLANTS_ is the EF for N_2_O from WWTPs (kg N_2_O—N / kg N).

**Table 3:** Selected values for the estimation of the nitrogen load in Switzerland based on the IPCC methods (IPCC 2006, 2019).

Variable	2006 IPCC guidelines coefficients for Switzerland	2019 refinement of the IPCC guidelines for Switzerland
P: Swiss protein consumption (t/year)	306,000	306,000
F_NPR_: Nitrogen conten of protein (default value) (kgN/kg protein)	0.16	0.16
T_PLANT_: Connection rate to WWTP (%)	97%	97%
F_IND-COM_: Factor for industrial and commercial protein (default value) (-)	1.25	1.25
F_NON—COM_: Factor for nitrogen in non-consumed protein disposed in sewer system (default value) (-)	–	1.09
N_HH_: Additional nitrogen from household products added to the wastewater (default value) (-)	–	1.08
Calculated total nitrogen load to WWTP (t N/year)	60,000	70,000

#### Approach b

The 2019 refinement of the 2006 IPCC guidelines (IPCC 2019) was applied according to Eq. (3) of this study. The values for the estimates were chosen from the Swiss implementation of the 2006 IPCC guidelines, and if not available, according to the default values in the 2019 refinement. The EF applied was 1.6 ± 0.5%, which was calculated as the mean and the standard error of the monitoring campaigns used for the guideline (Table S5, SI) (IPCC 2019). Numerical values of other variables are given in Table 3.

(3) $$N_{2}O_{PLANTS}=Protein\cdot \;F_{NPR}\cdot \;T_{PLANTS}\cdot N_{HH}\cdot F_{NON-COM}\cdot F_{IND-COM}\cdot EF_{PLANTS}$$

where N_2_O_PLANTS_ are the total, direct N_2_O emissions from WWTPs in Switzerland per year (kgN_2_O—N/year), Protein is Swiss protein consumption (kg protein/year), T_PLANT_ is the connection rate to WWTPs (-), F_NPR_ is the fraction of nitrogen in protein (-), N_HH_ is the additional nitrogen from household products added to the wastewater (-), F_NON—COM_ is the factor for nitrogen in non-consumed protein in the sewer system (-), F_IND-COM_ is the factor for industrial and commercial protein (-), and EF_PLANTS_ is the EF for N_2_O from WWTPs (kg N_2_O—N / kg N).

#### Approaches c and d

For the bottom-up approach, we used the data set described in (Strähl et al., 2013) to estimate the nitrogen load in the wastewater treated of all Swiss WWTPs. This dataset contains information on influent loads, treatment type and treatment performance for WWTPs covering ∼70% of the wastewater treated in CH in 2011 (Strähl et al., 2013). We assumed the same fractions of WWTP types and extrapolated the nitrogen loads found in the dataset to the WWTPs treating the remaining ∼30% of the wastewater in Switzerland. An overview of the data set is provided in section S6, SI. Countrywide emissions in approaches c and d were calculated according to Eq. (4). Approach (d) was calculated for two scenarios: d1 incorporates a high emission factor for the carbon removal category and d2 a low emission factor.

(4) $$N_{2}O_{PLANTS}=EF_{PLANTS}\cdot N_{PLANTS}$$

where N_2_O_PLANTS_ is the total, direct N_2_O emissions from WWTPs in Switzerland per year (kgN_2_O—N/year), EF_PLANTS_ is the estimated average EF of Switzerland (kg N_2_O—N / kg N) and N_PLANTS_ is the total nitrogen load treated in wastewater treatment plant (kgN/year).

The average EF was assessed by calculating the arithmetic mean of all monitoring campaigns reported in this study (approach c) or a weighted sum of emission factors estimated separately for the three categories of WWTPs (carbon removal, nitrification only, and nitrogen removal) in Switzerland (approaches d1 and d2) (Eq. (5)).

(5) $$EF_{PLANTS}=\sum_{i=1}^{3}f_{i}\cdot EF_{i}$$

where EF_PLANTS_ is the estimated average EF of Switzerland in scenarios (d1) and (d2) of approach (d) (kg N_2_O—N / kg N), f_i_ is the fraction of nitrogen loading in Switzerland treated in WWTPs belonging to category i, and EF_i_ is the estimated EF for category i.

#### Estimation of uncertainties

We estimated the uncertainties (standard deviation) of the estimated average EFs (*EF_PLANTS_*) using linear error propagation. The standard error (SE) of the countrywide EF estimates for approaches (b), (c), (d1) and (d2) were calculated by:

(6) $$SD_{EF}=\;\sqrt{\sum_{i=1}^{m}f_{i}^{2}{\left(\frac{\sigma_{i}}{\sqrt{n_{i}}}\right)}^{2}}$$

where SD_EF_ is the standard deviation of the estimated average EF, m is the number of categories of WWTPs (b,c: 1, d1, d2: 3), n_i_ is the number of samples in category i, f_i_ is the fraction of nitrogen loading in Switzerland treated in WWTPs belonging to category i, and σ_i_ is the standard deviation of the EFs in category i. The fraction $\frac{\sigma_{i}}{\sqrt{n_{i}}}$ represents the standard deviation of EF_i_.

Approach (a) is based on a single fixed EF, so uncertainty cannot be quantified.

## Results and discussion

### N_2_O emission factors from long-term monitoring campaigns

**Fig. 2:**
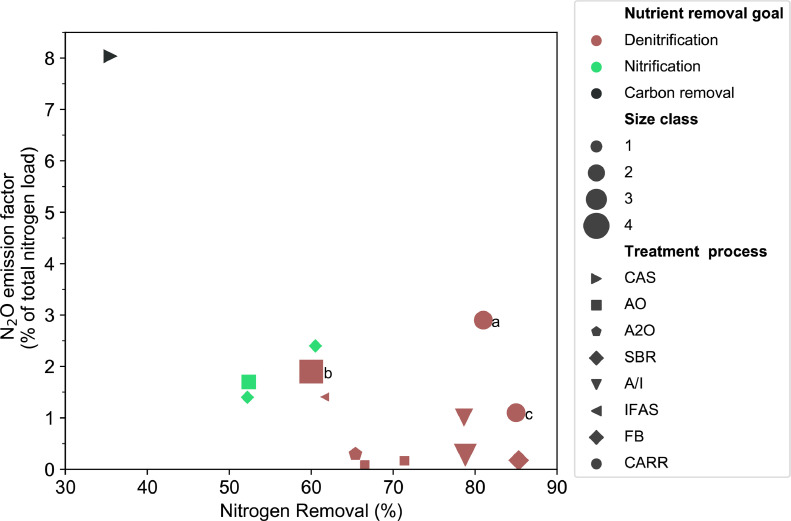
N_2_O EF calculated for all available monitoring campaigns as a function of the yearly average nitrogen removal efficiency. Colors describe nutrient removal goal. Small letters highlight campaigns assessed with a monitoring method other than the flux chamber method (a: (Daelman et al., 2015), b: (Kosonen et al., 2016), c: (Chen et al., 2019)). Size of data points describes the size class of the WWTPs (1: 〈 50,000 PE, 2: 50,000 – 200,000 PE, 3: 200,000 – 500,000 PE, 4: 〉 500,000 PE). Shape of data points indicates treatment process.

The monitoring campaigns studied showed a yearly N_2_O EF ranging from 0.1% to 8% of the total influent nitrogen load (Fig. 2). The range of EF is significantly wider than has been reported from previous long-term monitoring campaigns (1.1 to 2.9%), mainly due to the high EF of the Giubiasco WWTP, whose treatment goal is carbon removal. The average value of 1.6% for all studies is comparable to the value proposed in the updated IPCC guidelines (IPCC 2019). However, the high standard deviation of the average EF (2% equivalent to 125% of the mean value) shows clearly that using average EFs for countrywide extrapolations leads to very high uncertainties, which is in line with previous research (Valkova et al., 2020). Characterizing the N_2_O EF of a WWTP depending from key indicators is therefore essential for the robust calculation of countrywide EFs.

**Fig. 3:**
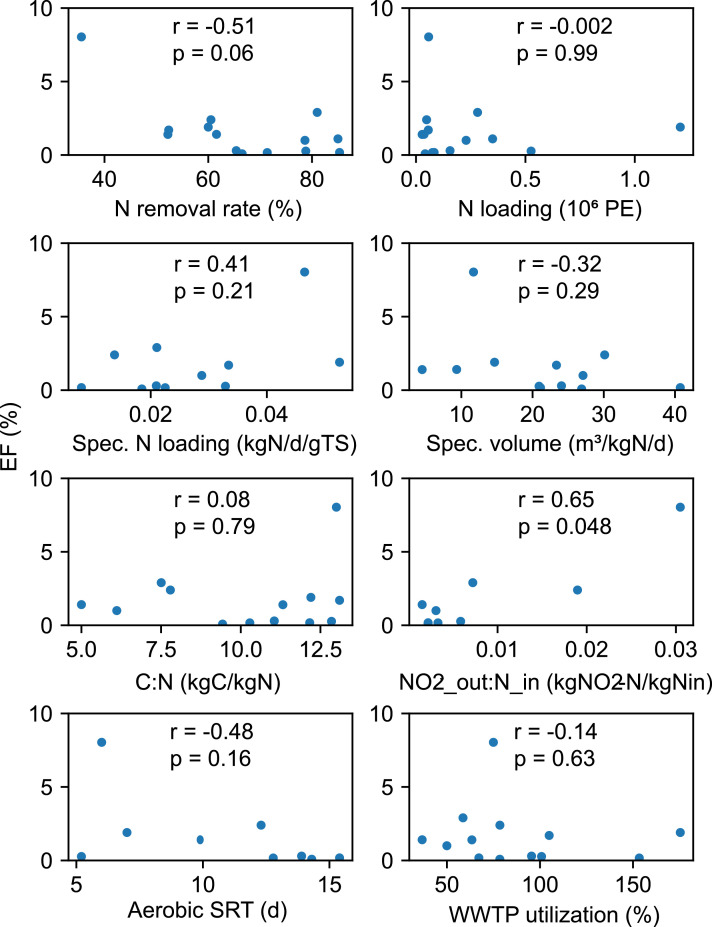
Correlation coefficients (spearman's rank, r) and significance levels (p) of selected variables with N_2_O EFs. Abbreviations: C:*N* = ratio in the influent; N_in = nitrogen inflow; *r* = correlation coefficient; Spec. = specific; SRT = sludge retention time.

None of the explanatory factors displayed in Fig. 2 exhibits a strong relation with the N_2_O EF. While nitrogen removal efficiency displays a weak and insignificant correlation (*r* = −0.51, *p* = 0.06, Fig. 3), treatment size does not correlate at all with N_2_O EF (*r* = −0.002, *p* = 0.99, Fig. 3). The low relevance of the reactor configuration and the treatment process can be only discussed qualitatively due to the low number of monitoring campaigns for different processes: Firstly, flow through activated sludge systems exhibited a wide range of EFs (0.08%−8%; *n* = 6). The systems monitored with our setup with an anoxic zone during the whole year (processes: AO and A2O, *n* = 3) did not exceed an EF of 0.3%. The very low EFs are not in line with the value (1.9%) for the Viikinmäki WWTP, which uses with an AO process. This dissimilarity may be explained by the difference between its influent conditions: Viikinmäki is served by a separated sewer system and the climate in Finland differs from that of central Europe (Kosonen et al., 2016). Moreover, the nitrogen removal efficiency of the biological treatment is 60%: quite low compared to the other AO and A2O systems (Fig. 2). Secondly, the EFs of two activated sludge plants with SBR configuration differed substantially (0.3% vs. 2.3%) as their nutrient removal goal differed: nitrification vs. denitrification. Previously, SBR systems were reported to cause generally higher N_2_O emissions (Vasilaki et al., 2019), but our results indicate that low EFs can be reached in SBR systems too. Thirdly, the two A/I activated sludge systems varied substantially (0.2% vs 0.8%) even though they shared the same removal goal, denitrification. Finally, both biofilm systems monitored had a high EF (1.4%), which is closely in line with previously reported values (Bollon et al., 2016). The systems monitored by Daelman et al. (2015) at Kralingseveer WWTP and Chen et al. (2019) at Avedøre WWTP have carrousel reactors for the biological treatment and exhibited high EFs (1.1% and 2.9% respectively).

The EFs assessed by other research groups are higher on average. This might be linked to the monitoring approach. In contrast to our flux chamber based monitoring approach, the other monitoring campaigns were conducted using i) measurement of dissolved N_2_O in the biological treatment combined with a stripping model and ii) the measurement of the collected off-gas of covered WWTP. The study by Chen et al. (2019) used approach i) and resulted in an EF similar to that found in this study. The studies by Daelman et al. (2015) and Kosonen et al. (2016) used approach ii) and reported very high EFs despite high nitrogen removal in the biological treatment.

Unlike the flux chamber approach, emissions from unaerated zones in the biological treatment and secondary clarification can be assessed using approaches i) and ii). However, the relative contribution of these emissions has been found to be of minor importance (Chen et al., 2019; Mikola et al., 2014). Three main sources of uncertainty arise when using the flux chamber method for monitoring N_2_O emissions from the aerated zone in the biological treatment. Firstly, inhomogeneous air supply within the biological treatment due to preferential flows of air and aging of the membrane aerators can locally affect airflow rates and calculated emissions substantially. To reduce those uncertainties, we used a multi-flux-chamber-approach with at least three flux chambers per lane to cover the full range of off-gas concentrations within a treatment lane. Secondly, inhomogeneity in nutrient and sewage loading of parallel lanes in a WWTP may contribute to variation in the emissions between lanes, which remains unobserved if not all lanes are monitored (Gruber et al. 2020). Thirdly, the quality of air flow and blower data differs highly between plants, ranging from blower frequency data of single blowers supplying several lanes to air flow meters installed in each zone of a treatment lane. To quantify the relevance of the uncertainties and compare the off-gas monitoring approaches, a study comparing all monitoring approaches applied on one WWTP is needed.

### Variables relevant for yearly N_2_O EF

Fig. 3 shows that the EF displays the highest and only statistically significant correlation with the effluent NO_2_^−^ load expressed as a fraction of the incoming nitrogen load. Similarly, concentration peaks coincided with N_2_O emissions peaks at WWTP with high EF, where NO_2_^−^ effluent concentrations were measured (Giubiasco, Lucerne, Kralingseveer). NO_2_^−^ accumulation has previously been linked to high N_2_O emissions in nitritation-denitrification systems (Kampschreur et al., 2008a; Peng et al., 2015). A negative correlation with nitrogen removal efficiency was expected, because denitrifying communities have a high capacity to scavenge N_2_O produced during both anoxic and aerobic conditions, which has been demonstrated in lab- and full-scale WWTP studies (Conthe et al., 2018; Rodriguez-Caballero et al., 2015). However, a few WWTPs (Lucerne, Avedøre and, most strikingly, Kralingseveer) had a high EF factor (≥ 1%) despite high nitrogen removal efficiency, most likely due to nitrite accumulation. Excluding the Kralingseveer WWTP data from the correlation analysis results in a strong and substantial relation between the EF and nitrogen removal efficiencies (*r* = 0.73, *p* = 0.005).

Other variables that could potentially be linked to N_2_O did not show significant correlation: nitrogen loading, specific reactor volume, C to N ratio at inflow, aerobic SRT, and WWTP utilization (average/design load). However, yearly average values are only partly useful for such correlation analysis, because seasonal peak phases are not well-represented (Vasilaki et al., 2018). Nevertheless, we conclude from the relations found that countrywide estimations can be improved by considering three factors governing the N_2_O EF of a WWTP: i) seasonal emission pattern and NO_2_^−^ accumulation, ii) all-year denitrification, and iii) unstable nitrification. These factors are discussed in the following sections.

### Seasonal emission pattern and NO_2_^−^ accumulation

**Fig. 4:**
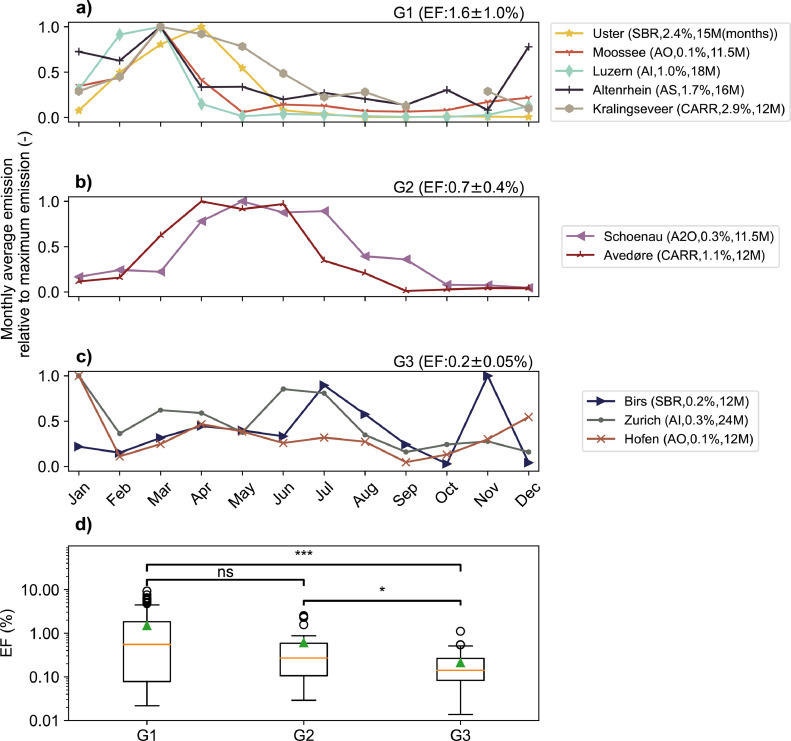
Monthly average N_2_O emission as a fraction of the monthly maximum emission for all monitoring campaigns analysed (a-c). The monitoring campaigns are grouped by the presence and shape of a seasonal emission pattern (G1: peak month in Feb/March, G2: peak months Apr/May-Jun/Jul, G3: no clear emission peak phase). EFs displayed above panels represent average EFs and standard deviation for each group. Boxplots (d) displaying the EFs in different groups (G1–3). First quartiles, medians (red line), and third quartiles are displayed in the box. Triangles indicate mean values and circles represent outliers. Significance levels (Mann-Whitney-Wilcoxon) indicates whether groups (G1 or G2) are significantly higher than pairing groups (G2 or G3) (not signficant (ns) > 0.05, * < 0.05, ** < 0.01, *** < 0.001).

A strong and reproducible emission pattern has been found in our own N_2_O emission monitoring campaign and in all previous studies except for the Viikinmäki study (Fig. 4). Emissions typically peaked in March, April or May and dropped over several months to a minimum in September or October. Hence, we analysed the seasonal emission pattern of the long-term monitoring campaigns for all biological nutrient removal (BNR) activated sludge processes in temperate climates, since this category represents the majority (*n* = 10) of the WWTPs monitored. The analysis of seasonality is considered representative, because for some WWTPs more than 1 year of data were available (Uster, Lucerne, Altenrhein) and the observations represent independent measurements of comparable processes during different years.

The seasonal emission pattern with a high emission peak in March or April was only apparent in five of the monitoring campaigns (Fig. 4, group G1, panel a). Two WWTPs exhibited a shifted pattern with extended peaks between April and July (group G2, panel b), and three WWTPs featured a rather uniform pattern with only occasional peaks (group G3, panel c). The average EF was significantly higher for the WWTPs exhibiting the seasonal EF than those without (Mann-Whitney-Wilcoxon: *U* = 3095, *p* = 6 × 10^−4^; Fig. 4, panel d). Only one of the WWTPs with an emission peak in March or April had an EF clearly below 1% (Moossee WWTP). WWTPs with a seasonal emission pattern emitted mostly between February and May. The high contribution of the emission peak phase to the total emissions highlights the strong need to better understand the causes of the seasonal emission pattern (Chen et al., 2019). Additionally, it signifies that emission reduction strategies should be based and tested on long-term monitoring campaigns covering at least 1 year (Duan et al., 2020). The reproducibility of the emission pattern on WWTPs with high EF (panel a, b) and between different lanes in a WWTP suggests that monitoring N_2_O emissions over 1 year is sufficient.

On several WWTPs (Lucerne, Uster, Kralingseveer), the pattern could be partly linked to NO_2_^−^ accumulation in biological treatment during the spring season. We hypothesize that reduced NO_2_^−^ oxidizing bacteria (NOB) performance could be the cause of seasonal NO_2_^−^ accumulation (Gruber et al., 2021; Vieira et al., 2018) and lead to N_2_O peak emissions induced by enhanced nitrifier denitrification or incomplete heterotrophic denitrification. The emission pattern and the emission peaks in spring help to prioritize efforts to mitigate N_2_O emissions, but the high impact on the yearly EF is problematic for a countrywide extrapolation based on only a few monitoring campaigns, since the frequency of WWTPs with seasonal peaks is unknown. Additionally, NO_2_^−^ effluent concentrations do not fully represent the NO_2_^−^ concentrations in the reactors, because NO_2_^−^ can accumulate locally in a WWTP or even within sludge flocs (Chen et al., 2018). As a consequence, monitoring of N_2_O emission patterns at WWTPs with plug-flow characteristics or multiple biological treatment steps, such as the Kralingseveer WWTP, may be less accurate if effluent concentrations are used or if concentrations are sampled only at one location. For such cases, extensive monitoring of nitrogen species at a range of locations may be advantageous. In order to avoid NO_2_^−^ accumulation, all-year denitrification appears to be an efficient strategy. WWTPs without seasonal emission patterns (Birs, Zurich and Hofen) or with low EFs (Moossee) had a very low proportion of NO_2_^−^ in the effluent of the biological treatment (0.2–0.5% relative to the yearly nitrogen influent load). All of these WWTPs practise all-year denitrification.

### Year-round denitrification and N_2_O reduction

**Fig. 5:**
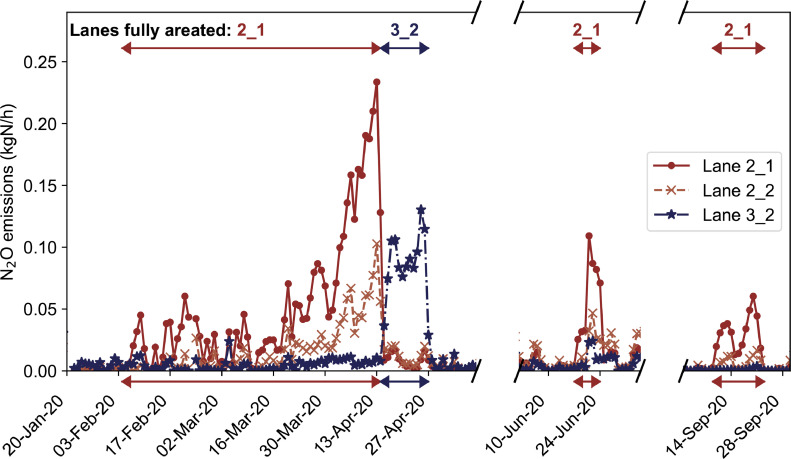
N_2_O emission at Hofen WWTP between January and October 2020. Arrows indicate the lanes where all three compartments were fully aerated.

To test the seasonally varying influence of an anoxic zone and denitrification on N_2_O emissions, full-scale tests were performed at the Hofen WWTP. Some WWTPs in Switzerland are operated for nitrogen removal year-round whereas other treatment plants are fully aerated in winter, and thus only perform denitrification in summer. The responses of N_2_O emissions to switching aeration on or off in the first compartment of the biological treatment train are shown in Fig. 5. The N_2_O emissions were substantially increased on the fully aerated lanes (lanes 2–1 and 3–2). Amongst the monitored lanes sharing the same secondary clarifier, the one with an anoxic zone (2–2) had lower emissions than the fully aerated one (2–1) but still had higher emissions than the lane with anoxic zones on both lanes sharing the same secondary clarifier (lane 3–2). When the conditions were swapped and lane 3–2 was fully aerated, the emission pattern reacted immediately and switched completely. The anoxic zone, however, does not always have such a substantial impact on the emissions. During the first phase of the first experiment (beginning of February to mid-March), emissions on lanes 2–1 and 2–2 only slightly increased. Additionally, the emissions rose to a lower level in June and only marginally in September. The importance of denitrification to reaching low emissions highlights the need to consider nitrogen removal rates in countrywide estimations of N_2_O emissions.

The varying impact of an anoxic zone underpin our conclusion that the emission peaks in spring are caused by imbalanced nitrification and NO_2_^−^ accumulation. An anoxic zone prevents NO_2_^−^ and N_2_O accumulation probably via the return sludge, since both intermediates can be reduced by denitrification as long as sufficient organic substrate is available (Pan et al., 2013). The seasonal dependence of denitrification on the emissions might be linked to the yearly variation of the microbial community and nitrifiers in a WWTP and relates to the previous discussion about yearly NOB variation (Griffin and Wells 2017; Gruber et al., 2021; Ju et al., 2014). Experimental support for the relevance of seasonal nitrifier variation can be found in the monitoring campaign at the Giubiasco WWTP, which represents an extreme case of unstable nitrification and highlights its impact on N_2_O emissions.

### Unstable nitrification and N_2_O emission pattern

**Fig. 6:**
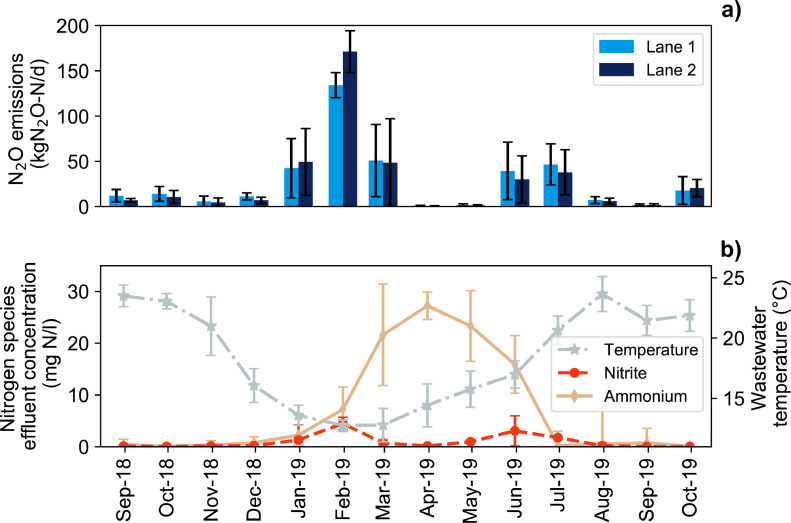
Monthly average N_2_O emissions measured for both lanes at the Giubiasco WWTP (panel a) and wastewater temperature, monthly average NH_4_^+^-N, and NO_2_^−^-N effluent concentrations (panel b). Error bars indicate standard deviation of daily averaged values.

To characterize the risk of high N_2_O emissions in a process with unstable nitrification, we analysed the Giubiasco WWTP in more detail. The Giubiasco WWTP is a CAS system with carbon removal as a treatment goal. The N_2_O emission pattern exhibited a strong seasonal variation (Fig. 6a). As a consequence of higher temperatures, nitrifiers can actually proliferate during the summer months and a seasonal occurrence of nitrification is the result at Giubiasco WWTP (Fig. 6b). The yearly emission pattern exhibits two emission peak phases (January – March & June - July): these coincide with a dramatic change in nitrification performance. Nitrification did not occur during winter, at low temperatures. Therefore, the ammonium effluent concertation of the WWTP exhibits a pattern that is opposite of the wastewater temperature curve. During the transition from a nitrifying to a non-nitrifying process, the NO_2_^−^ concentrations in the WWTP effluent increased and massive amounts of N_2_O were emitted.

The high EF (8%) for a non-BNR system contrasts strongly with previous studies and the IPCC guidelines, which suggests that a high EF is linked to uncontrolled and incomplete nitrification. In most previous studies, non-BNR systems were found to have low emissions (Vasilaki et al., 2019). Our study demonstrates that long-term monitoring is required to assess EFs from non-BNR systems, unless nitrification can be excluded all year. Most of the previously reported monitoring campaigns were conducted over short periods, but a few discontinuous long-term monitoring campaigns have been conducted for non-BNR system. A study on a Brazil WWTP over 1 year resulted in a very low EF of 0.12% (Brotto et al., 2015). The nitrogen removal efficiency and nitrification exhibited a strong seasonal variation, but NO_2_^−^ was comparatively low (0–0.8 mg NO_2_^−^-N/L) in all effluent samples. A possible explanation for the differences from the Giubiasco WWTP is the tropical climate, which led to very high wastewater temperature (24 °C at minimum). High emissions for WWTPs with variable nitrogen removal efficiencies were reported from three monitoring campaigns using a discontinuous monitoring approach over a year (Yan et al., 2014). In that study, a WWTP with similarly low nitrogen removal efficiencies as the Giubiasco WWTP had a very high average EF of 3.6%. The yearly temperature patterns were very similar to the WWTPs monitored in our study.

Further continuous, long-term monitoring campaigns on carbon removal WWTPs are essential to provide better estimates of EF variability. We expect negligible EFs from carbon removal systems that completely avoid BNR, partly explaining the low EF of previous studies. For emission mitigation, limiting the SRT to prevent nitrifier growth could be a feasible strategy for carbon removing plants. However, the strategy leads to impaired removal of organic compounds in the biological treatment (Falas et al., 2016) and, thus, to a trade-off decision between effluent quality and the carbon footprint of a WWTP.

### Countrywide extrapolation of N_2_O emissions and uncertainties

**Fig. 7:**
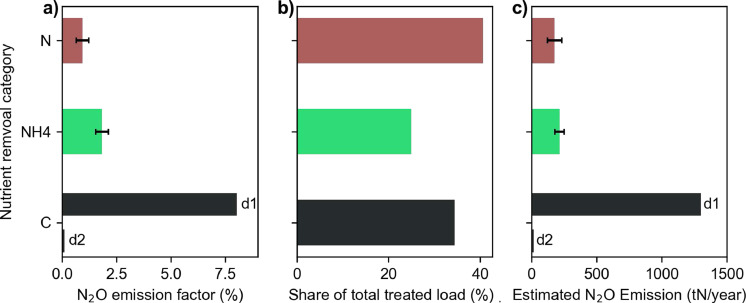
Estimation of the countrywide EF for Switzerland (CH). *N* = plants with an anoxic zone for year-round denitrification; NH4 = nitrifying plants denitrifying only during the warm season; *C* = plants not required to nitrify. C (d1) and (d2) denote EFs for the two scenarios tested. Error bars indicate the standard errors. (a) Estimated EFs per nutrient removal category. (b) Treated nitrogen load per nutrient removal categories in CH in 2011. (c) Estimate emissions for each category assuming activity data in Table 4.

The dependencies of the N_2_O EF discussed above and the corresponding understanding of the mechanistic processes derived from the 14 long-term monitoring campaigns on eight types of WWTP processes led us to propose a refined approach to estimating on-site N_2_O emissions of WWTPs in Switzerland. We suggest calculating an EF for the three nutrient removal categories of carbon removal, nitrification only, and year-round nitrogen removal with the overall assumption that a lower nitrogen removal results in higher emissions and a higher probability of NO_2_^−^ accumulation unless nitrification can be excluded completely. We calculated an average EF for Switzerland by multiplying the average EF for each category (Fig. 7a) with the share of nitrogen load treated by each category (Fig. 7b), (Table 4). The total emissions were calculated for two scenarios with our approach (d1 and d2), because data availability for carbon removal plant was not sufficient and emissions were expected to differ substantially between such WWTPs. We did not propose a linear regression model for EF extrapolation, since data on effluent NO_2_^−^, the best predictor for EFs (Fig. 3), was limited on a countrywide level in Switzerland, and is in most other countries.

**Table 4:** Estimation of direct N_2_O emissions from wastewater treatment plants in Switzerland using five calculation methods.

Estimation approach	Activity data (nitrogen load) (tN/year)	EF_PLANTS_ (Estimated average EF) (%)	N_2_O_PLANTS_ (Total emissions and uncertainties) (tN_2_O—N/year)
a (2006 guidelines)	60,000	0.032	19
b (2019 refinement)	70,000	1.6 ± 0.4	1120±280
c (Swiss specific activity data, average EF)	47,000	1.6 ± 0.5	744±230
d1 (Swiss specific activity data, weighted EF, Carbon removal: high EF)	47,000	3.6 ± 0.2	1690±90
d2 (Swiss specific activity data, weighted EF, Carbon removal: low EF)	47,000	0.9 ± 0.2	410±90

The results of the new methods (approaches d1 and d2) are compared with the current IPCC approaches (approaches a and b) and an average EF of all 14 EFs (approach c) discussed in this study (Table 4). The estimated total nitrogen load to WWTP based on our bottom-up approach (c, d) is lower than that calculated with the IPCC approaches (a, b). Nevertheless, the emission estimates calculated with the 2006 guidelines are drastically lower than the other estimates due to the very low EF applied (Table 4). N_2_O emissions calculated with our approaches (c, d1, d2) vary substantially and depend primarily on the estimated EF for carbon removal plants (Fig. 7c). The 2019 refinements are in the range of the total emissions estimated with our approaches and data. In summary, all the estimates show that direct N_2_O emissions from WWTPs are important and sum up to 0.3–1.4% of total Swiss GHG emissions (∼50 Mt_CO2,eq_/year in 2011) (FOEN 2020). Notably, these estimations do not include N_2_O emissions originating from the effluent of the WWTP after discharge to the environment and the total GHG emission in Switzerland in 2011 were around 10% higher than in 2018. Accordingly, we expect an increasing importance of N_2_O emissions from WWTPs to the total Swiss GHG emissions, especially assuming the projected, continued decline of total GHG emissions.

The discrepancies between the activity data from the IPCC approaches and our data confirm that a country-specific bottom-up approach should be prioritized for estimation if sufficient data is available (Ramirez-Melgarejo et al., 2020). The low emission estimates yielded from the 2006 guidelines highlight that an update of the estimation methods was clearly required for more accurate representation of wastewater treatment in GHG inventories. However, our results show that the 2019 refinement is not optimal for calculating country specific EFs for two reasons: (i) The selection of monitoring campaigns in the guidelines used to calculate the average EF is arbitrary and not necessarily representative for a country. (ii) The EFs applied (section S5, SI) originate mostly from short-term grab sampling, which does not provide representative EFs (Daelman et al., 2013). Methods building on a country-specific representation of wastewater treatment, such as ours, not only yield more accurate estimations but are better suited to resolve changes in wastewater treatment efficiencies over time (Figure S4). An increase of the nitrogen removal efficiencies in wastewater treatment plants over the last decades, as reported in most developed countries due to increased effluent requirements, could be implemented with our approach to obtain representative emission time series (Van Drecht et al. 2009). The expected change in emissions over time could thus be quantified. As required by the Kyoto and Paris protocols (United Nations climate change 1997, 2015), the reporting of GHG emissions to the UNFCCC (United Nations climate change 1992) always refers back to 1990 and representative emission time series are therefore considered important.

We conclude that identifying robust predictors for EFs from WWTPs is of high importance to making reliable predictions for the total N_2_O emission from wastewater treatment. Previously, linear regression modeling was proposed by Valkova et al. (2020) as a versatile and precise predictor for countrywide N_2_O emissions from WWTPs. Their study was based on short-term monitoring campaigns over a few weeks, which does not provide representative EF estimation, and reported substantially lower EFs (0–1.5%) than the values reported in our study and other long-term monitoring campaigns (Chen et al., 2019). Moreover, a linear relation between nitrogen removal efficiency and EF cannot be assumed (Fig. 3 and potentially low EFs for carbon removal WWTPs). Our approach is based on long-term monitoring data and on grouping of EFs according to meaningful characteristics of WWTPs, which additionally reduces the uncertainties of EFs (Table 4). However, a clear need to better characterize emissions from carbon removal WWTPs has been identified. Estimates of the proportion and EFs of carbon removal WWTPs have to be made by expert judgements until more monitoring data is available. A fourth nutrient removal category could then be implemented in our method to differentiate between the two types of carbon removal WWTPs and combine scenarios (d1) and (d2). Ultimately, we believe that our approach can be used for N_2_O emission estimations in other countries with temperate climates and significantly improve the representativity of emission estimation over the IPCC approach. For the extrapolation to countries in other climate zones, continuous, long-term monitoring campaigns in those climate zones are absolutely necessary.

## Conclusions

N_2_O EFs from WWTPs exhibit a strong relation with the effluent NO_2_^−^ (compared to the influent nitrogen load) and nitrogen removal efficiency. Since data availability for NO_2_^−^ effluent loads on a countrywide level is usually limited, we suggest calculating a countrywide EF from the weighted, average EFs of three nutrient removal categories carbon removal, (EF: 0.1–8%), nitrification only: (1.8%), and full nitrogen removal (0.9%). The overall assumption of the approach is that categories with higher nitrogen removal result in lower emissions unless nitrification is not active at all. The approach allows representative, country-specific estimations of the N_2_O emissions from WWTPs. Applied to Switzerland, the estimations result in an average EF of 0.9–3.6% and total emissions of 410–1690 tN_2_O-N/year, which corresponds to 0.3–1.4% of total Swiss GHG emissions.

Uncontrolled nitrification in WWTPs with carbon removal as a treatment goal can cause very high emissions coupled with NO_2_^−^ accumulation during wash-out and re-growth of nitrifiers. Partial nitrification should be avoided by stringent SRT control, because increased N_2_O emissions can be excluded, as long as nitrification is not present in a WWTP. Consequently, uncertainties linked to the high variability of carbon removal WWTPs (estimated EFs: 0.1–8%) have to be considered in countrywide emission estimations. Further continuous, long-term monitoring campaigns on carbon removal WWTPs are required to reduce the uncertainties of EF estimates. Yearlong continuous monitoring campaigns are essential to assess representative EFs given the high temporal variability encountered in 14 long-term monitoring campaigns. Yearly N_2_O emissions patterns for activated sludge based WWTPs with high EFs (>0.5%) are very dynamic and comparable among different WWTPs and lanes of the same WWTP with separated sludges. Hence, assuming yearly reproducibility of EFs is justified for a particular WWTP. The emission peak phase often coincides with NO_2_^−^ accumulation in the biological treatment. We expect that limiting NO_2_^−^ accumulation is the key factor reducing N_2_O emissions in WWTPs.

WWTPs with year-round denitrification often exhibit low EFs and rather uniform emission pattern. Introducing anoxic conditions at the beginning of the biological treatment immediately reduces N_2_O emissions over the whole biological treatment, including the aerobic zones. However, fully aerobic conditions do not always result in high N_2_O emissions.

## Author contributions

W.G., L.v.K., L.B., D.B., E.M. and A.J. designed the study. All authors provided helpful feedback and suggestions throughout work on the study. L.v.K., L.B. and W.G. were responsible for data collection of process performance data and monitoring data. L.V., L.v.K., W.G. and R.L. programmed the routines to evaluate the monitoring data. L.B., D.B., L.v.K., W.G., A.M., M.K., L.V., N.K., and A.J. planned and conducted the monitoring campaigns. K.F., L.v.K., M.L., R.L., and W.G. analyzed the data. The manuscript was written by W.G with helpful reviews from A.J., L.v.K., D.B., and E.M.

## Declaration of Competing Interest

The authors declare that they have no known competing financial interests or personal relationships that could have appeared to influence the work reported in this paper.

## Appendix Supplementary materials

Image, application 1
