# Supplementary material for: Estimation of countrywide N2O emissions from wastewater treatment in Switzerland using long-term monitoring data
Source: Water Res X. 2021 Sep 30;13:100122. doi: 10.1016/j.wroa.2021.100122 (PMC8503907; doi:10.1016/j.wroa.2021.100122)
Supplement: Supplementary file 1 [file mmc1.docx]

# Supporting Information: Estimation of countrywide N_2_O emissions from wastewater treatment in Switzerland using long-term monitoring data

Wenzel Gruber^a,b,^*, Luzia von Känel^b^, Liliane Vogt^a,b^, Manuel Luck^a,b^, Lucien Biolley^b^, Kilian Feller^a^, Andrin Moosmann^a^, Nikita Krähenbühl^a^, Marco Kipf^a^, Reto Loosli^b^, Michael Vogel^b^, Eberhard Morgenroth^a,b^, Daniel Braun^b^, Adriano Joss^a^

* Corresponding author

^a^ Eawag, Swiss Federal Institute for Aquatic Science and Technology, 8600 Duebendorf, Switzerland

^b^ Institute of Environmental Engineering, ETH Zürich, 8093 Zürich, Switzerland

## Monitoring setup

A detailed description of the monitoring setup is currently available under the following link:

<https://doi.org/10.25678/0003WD>

The description contains the following elements:

- Description of the system
- P&ID of the system
- Wiring diagram
- Components list
- Software for control and operation
- Technical drawings

## Monitoring campaigns

For each of the newly conducted monitoring campaigns, three files are attached as Supporting information: 1) a data sheet (Data_sheet_*WWTP*.pdf) with information on the biological treatment, on the origin of the lab data, and on the monitoring campaign, 2) a lab data sheet (*WWTP*_DATA_Lab.csv) with measured or estimated influent and effluent loads for total nitrogen and COD, and 3) a data sheet (*WWTP*_DATA_Emissions.csv) with highly resolved N_2_O emissions.

The three types of data files described exist for the monitoring campaigns on the following WWTP:

- Bazenheid
- Birs
- Giubiasco
- Hofen
- Moossee
- Schönau
- Werdhölzli

All data is available under the following link: <https://doi.org/10.25678/0003XE>

## Data availability

Table S1 Duration of monitoring campaigns and number of days evaluated (i.e. availability of the monitoring device).

| WWTP (location name) | Duration of the campaign (days) | Days evaluated (share of total) |
| --- | --- | --- |
| Bazenheid | 492 | 389 (80%) |
| Birs | 369 | 236 (64%) |
| Giubiasco | 382 | 325 (85%) |
| Hofen | 419 | 375 (90%) |
| Moossee | 248 | 345 (72%) |
| Schönau | 312 | 348 (90%) |
| Werdhölzli | 632 | 728 (87%) |

## Nitrogen mass balance of a primary clarifier


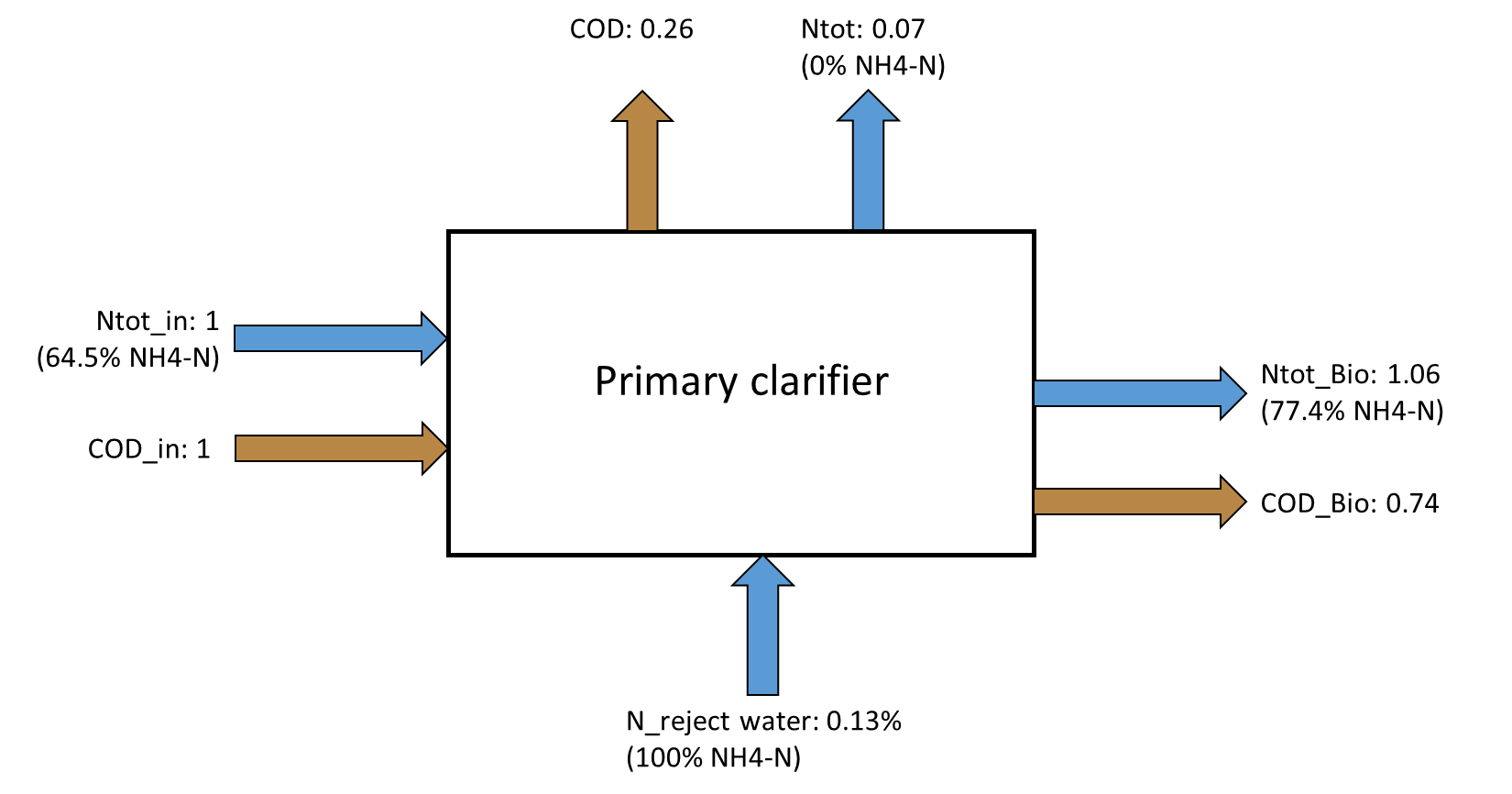


Figure S1 Nitrogen and COD mass balance of model primary clarifier based on (Gujer 2007) to estimate missing values for monitoring campaigns. Numeric values denote fractions of Ntot_in and COD_in. Percentage values below nitrogen values denote ammonium share of total nitrogen.

## Meta Data for correlation analysis

Table S2 Design characteristics of the WWTP. Abbreviations: PE = person equivalents, SRT = sludge retention time, N = nitrification and denitrification all year, NH4 = nitrification without requirement to denitrify all year, C = carbon removal. A/I = alternatingly fed and intermittently aerated, CAS = conventional activated sludge, BF = fixed bed biofilter, IFAS = integrated fixed film activated sludge, AO = anoxic oxic activated sludge, A2O = anaerobic, anoxic, oxic activated sludge, CARR = caroussel treatment.

| **Name** | **Design load**  **(PE)** | **Nutrient  removal goal**  **(-)** | **Process biological treatment (-)** | **Biomass type**  **(-)** | **Volume  biological treatment**  **(m^3^)** | **Volume secondary clarification (m^3^)** | **Total Volume biological treatment**  **(m^3^)** | **Average TS biological  treatment  (gTS/l)** | **Aerobic SRT**  **(d)** | **SRT**  **(d)** | **Supernatant  treatment**  **(-)** | **Co-digestion**  **(-)** | **Sandfilter**  **(-)** |
| --- | --- | --- | --- | --- | --- | --- | --- | --- | --- | --- | --- | --- | --- |
| Luzern | 280000 | N | A/I | AS | 36000 | 38400 | 74400 | 2.65 | 5.2 | 12 | No | No | No |
| Zurich | 670000 | N | A/I | AS | 60000 | 72000 | 132000 | 3.2 | 5.2 | 10 | Yes | Yes | No |
| Altenrhein_AS | 120000 | NH4 | CAS | AS | 8220 | 7500 | 15720 | 2.45 | 10 | 10 | No | Yes | Yes |
| Altenrhein_FB | 120000 | N | FB | BF | 1512 | 0 | 1512 | - | - | - | No | Yes | Yes |
| Uster | 48000 | NH4 | SBR | AS | 17472 | 0 | 17472 | 2.41 | 12.3 | 23 | No | No | Yes |
| Bazenheid | 50000 | N | IFAS | BF | 4112 | 0 | 4112 | - | 5.3 | 5.3 | Yes | Yes | No |
| Giubiasco | 100000 | C | CAS | AS | 6200 | 1904 | 8104 | 2.4 | 6 | 6 | No | No | No |
| Moossee | 50000 | N | AO | AS | 8940 | 4480 | 13420 | 3.02 | 14.3 | 21.7 | No | No | No |
| Schoenau | 180000 | N | A2O | AS | 27900 | 17000 | 44900 | 3.19 | 13.9 | 19.5 | Yes | No | Yes |
| Birs | 150000 | N | SBR | AS | 40500 | 0 | 40500 | 3.04 | 15.4 | 25 | No | No | No |
| Hofen | 50000 | N | AO | AS | 9000 | 9900 | 18900 | 4.42 | 12.78 | 19 | No | No | No |
| Kralingseveer | 360000 | N | CARR | AS | 39100 | - | - | 4.1 | - | - | No | - | No |
| Avedøre | 350000 | N | CARR | AS | - | - | - | 2.3 | - | - | - | - | - |
| Vikinmäkki | 840000 | N | A2O | AS | 92000 | 120600 | 212600 | 3 | 7 | - | No | - | - |

Table S3 Inflow and effluent characteristics. Abbreviations: Q = water flow, COD = chemical oxygen demand, TN = total nitrogen, NO_2_-N = nitrite load; in = influent, out = effluent, in_bio = after primary clarifier, out_bio = effluent biological treatment, out_WWTP = final effluent, Altenrhein_AS = activated sludge plant in Altenrhein, Altenrhein_FB = fixed bed plant in Altenrhein

| **Name** | **Q_in  (m^3^/d)** | **COD_in  (kg/d)** | **COD_out  (kg/d)** | **TN_in WW  (kg/d)** | **TN_in_DigSup  (kg/d)** | **TN_tot (kg/d)** | **TN_in_bio  (kg/d)** | **TN_out_bio  (kg/d)** | **TN_out_WWTP  (kg/d)** | **NO_2_-N out_bio (kg/d)** |
| --- | --- | --- | --- | --- | --- | --- | --- | --- | --- | --- |
| Luzern | 93’942 | 16’783 | 1576 | 2554 | 349 | 2903 | 2747 | 620 | 620 | 9 |
| Zurich | 172’699 | 81’078 | 3181 | 6869 | 0 | 6869 | 6311 | 1456 | 1456 | 37 |
| Altenrhein_AS | 18’436 | 8’816 | - | 462 | 170 | 632 | 673 | 398 | 398 | - |
| Altenrhein_FB | 8’816 | 3’804 | - | 231 | 85 | 316 | 336 | 151 | 151 | - |
| Uster | 15’973 | 4’519 | 249 | 527 | 170 | 697 | 580 | 229 | 205 | 11 |
| Bazenheid | 4’897 | 2’200 | - | 216 | 224 | 440 | 440 | 169 | 169 | 1 |
| Bellinzona | 17’559 | 8’996 | 478 | 714 | 83.1 | 797 | 692 | 446 | 446 | 21 |
| Moossee | 13’177 | 4’705 | 251 | 453 | 75 | 528 | 499 | 167 | 167 | 1 |
| Schoenau | 56’099 | 20’622 | 731 | 1997 | 0 | 1997 | 1866 | 691 | 691 | - |
| Birs | 24’029 | 12’090 | 165 | 812 | 250 | 1062 | 994 | 156 | 156 | 2 |
| Hofen | 25’387 | 8’788 | 493 | 825 | 187 | 1012 | 959 | 279 | 297 | 2 |
| Kralingseveer | 88000 | 25327 | 3380 | 3216 | - | - | 3375 | 963 | 963 | 24 |
| Avedøre | 70000 | - | - | - | - | - | - | - | 308 | - |
| Viikinmäki | 289900 | 176’839 | 12400 | 12877 | - | - | 14495 | - | 1160 | - |

Table S4 Correlation Data

| **Name** | **N_2_O EF**  **(%)** | **Average load**  **(PE of nitrogen load)** | | **Specific reactor volume biological treatment**  **(kg N/m^3^)** | | **Nitrogen**  **removal efficiency**  **biological treatment**  **(%)** | | **C to N ratio**  **(C2N)**  **(kgO_2_/kgN)** | | **Nitrite in effluent as share of total nitrogen**  **(%)** | | **Specific nitrogen loading of biological treatment**  **(kgN/(kgTS*d))** | | **Aerobic SRT**  **biological treatment**  **(d)** | | **WWTP utilization**  **(Average load/design load)**  **(%)** | |
| --- | --- | --- | --- | --- | --- | --- | --- | --- | --- | --- | --- | --- | --- | --- | --- | --- | --- |
| Luzern | 1 | 228917 | 27.1 | | 78.6 | | 6.1 | | 0.3 | | 0.029 | | - | | 49.9 | |  |
| Zurich | 0.3 | 525917 | 20.9 | | 78.8 | | 12.8 | | 0.6 | | 0.033 | | 5.2 | | 100.8 | |  |
| Altenrhein_AS | 1.7 | 56083 | 23.4 | | 52.4 | | 13.1 | | - | | 0.033 | | 10 | | 105.0 | |  |
| Altenrhein_FB | 1.4 | 28000 | 4.5 | | 52.2 | | 11.3 | | - | | - | | - | | 63.4 | |  |
| Uster | 2.4 | 48333 | 30.1 | | 60.5 | | 7.8 | | 1.9 | | 0.014 | | 12.3 | | 78.5 | |  |
| Bazenheid | 1.4 | 36667 | 9.3 | | 61.6 | | 5.0 | | 0.2 | | - | | 5.3 | | 36.7 | |  |
| Bellinzona | 8.0 | 57667 | 11.7 | | 35.5 | | 13.0 | | 3.0 | | 0.047 | | 6.0 | | 75.0 | |  |
| Moossee | 0.1 | 41583 | 26.9 | | 66.5 | | 9.4 | | 0.2 | | 0.018 | | 14.3 | | 78.4 | |  |
| Schoenau | 0.3 | 155500 | 24.1 | | 65.4 | | 11.1 | | - | | 0.021 | | 13.9 | | 95.5 | |  |
| Birs | 0.2 | 82833 | 40.7 | | 85.3 | | 12.2 | | 0.2 | | 0.008 | | 15.4 | | 67.2 | |  |
| Hofen | 0.1 | 79917 | 19.7 | | 72.4 | | 9.2 | | 0.2 | | 0.024 | | 12.8 | | 146.5 | |  |
| Kralingseveer | 2.9 | 281250 | - | | 81.0 | | 7.5 | | 0.7 | | 0.021 | | - | | 58.6 | |  |
| Avedøre | 1.1 | 350000 | - | | 85.0 | | - | | - | | - | | - | |  | |  |
| Viikinmäki | 1.9 | 1207917 | 14.7 | | 60.0 | | 12.2 | | - | | 0.053 | | 7.0 | | 175.4 | |  |

## Countrywide extrapolation of N_2_O emissions and uncertainties

Table S5 Monitoring campaigns and emission factors applied in the 2019 IPCC guidelines

| **Type of reatment process^1^** | **Categories** | **References** | **N_2_O emission factor (kg N_2_O-N/kg N)** | **Monitoring strategy (sampling, duration)** |
| --- | --- | --- | --- | --- |
| AO | BNR | (Daelman et al. 2015) | 0.028 | Continous, long-term |
| AO | BNR | (Foley et al. 2010) | 0.021 | Grab, short-term |
| AO | BNR | (Foley et al. 2010) | 0.045 | Grab, short-term |
| A2O | BNR | (Foley et al. 2010) | 0.013 | Grab, short-term |
| SBR | BNR | (Foley et al. 2010) | 0.023 | Grab, short-term |
| OD | BNR | (Foley et al. 2010) | 0.0080 | Grab, short-term |
| IA | BNR | (Kimochi et al. 1998) | 0.0005 | Grab, short-term |
| EA | BNR | (Foley et al. 2010) | 0.015 | Grab, short-term |
| A2O | BNR | (Wang et al. 2016) | 0.013 | Grab, short-term |
| CAS | BNR | (Aboobakar et al. 2013) | 0.00036 | Continous, short-term |
| AO | BNR | (Rodriguez-Caballero et al. 2014) | 0.12 | Continous, short-term |
| OD | BNR | (Masuda et al. 2018) | 0.00016 | Grab, short-term |
| AO | BNR | (Masuda et al. 2018) | 0.0013 | Grab, short-term |
| AO | BNR | (Masuda et al. 2018) | 0.0049 | Grab, short-term |
| Separate-stage BNR | BNR | (Ahn et al. 2010) | 0.00019 | Grab, short-term |
| Bardenpho | BNR | (Ahn et al. 2010) | 0.0036 | Grab, short-term |
| Step-feed BNR | BNR | (Ahn et al. 2010) | 0.011 | Grab, short-term |
| MLE | BNR | (Ahn et al. 2010) | 0.0007 | Grab, short-term |
| MLE | BNR | (Ahn et al. 2010) | 0.0006 | Grab, short-term |
| OD | BNR | (Ahn et al. 2010) | 0.0003 | Grab, short-term |
| Step-feed BNR | BNR | (Ahn et al. 2010) | 0.015 | Grab, short-term |
| Step feed, plug flow | BNR | (Ni et al. 2015, Pan et al. 2016) | 0.019 | Continous, short-term |
| SBR | BNR | (Bao et al. 2016) | 0.029 | Grab, short-term |
| SBR | BNR | (Rodriguez-Caballero et al. 2015) | 0.038 | Continous, short-term |
| Plug flow | Non-BNR | (Ahn et al. 2010) | 0.004 | Grab, short-term |
| Plug flow | Non-BNR | (Ahn et al. 2010) | 0.0062 | Grab, short-term |
| Step-feed non-BNR | Non-BNR | (Ahn et al. 2010) | 0.0018 | Grab, short-term |
| Plug flow | Non-BNR | (Masuda et al. 2015) | 0.023 | Grab, short-term |
| AO | Non-BNR | (Bao et al. 2016) | 0.013 | Grab, short-term |
| IA | Non-BNR | (Mello et al. 2013) | 0.0016 | Grab, short-term |
| ^1^ AO, Anaerobic-oxic activated sludge process; A2O, Anaerobic-anoxic-oxic activated sludge process; SBR, Sequencing batch reactor; OD, Oxidation ditch; IA, Intermittent aeration process; EA, Extended aeration process; CAS, Conventional activated sludge process; MLE, Modified Ludzack-Ettinger; BNR, biological nutrient elimination. | | | | |

## Nitrogen loads to Swiss WWTPs


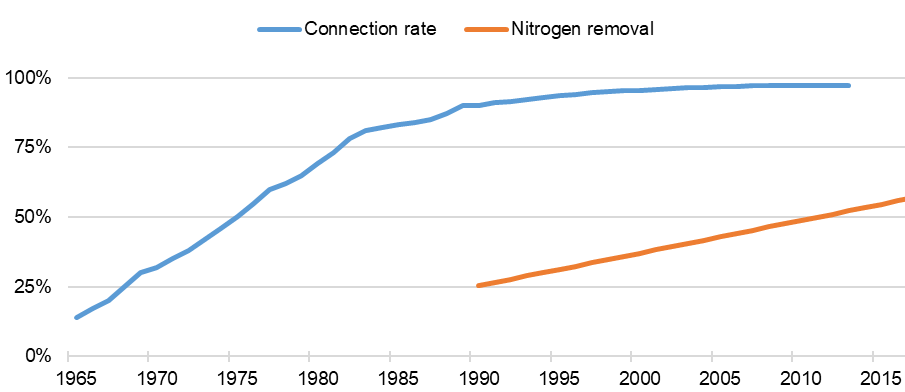


Figure S2 Connection rate of the Swiss population to WWTP over time and interpolated nitrogen removal.

Figure S3 Size distribution of Swiss WWTPs in the year 2011 with respect to number of plants and persons treated. Cat. I: <50’000 PE; Cat. II: 50’000 - 200’000 PE; Cat. III: 200’000 to 500’000 PE; Cat. IV: >500’000 PE.

Figure S4 Amount of person equivalents treated in Swiss WWTPs in 2011 depending on the treatment goal. Abbreviations: C = carbon removal; NH4 = nitrification without requirement to denitrify all year; N = nitrify and denitrify all year.

# References

Aboobakar, A., Cartmell, E., Stephenson, T., Jones, M., Vale, P. and Dotro, G. (2013) Nitrous oxide emissions and dissolved oxygen profiling in a full-scale nitrifying activated sludge treatment plant. Water Res 47(2), 524-534.

Ahn, J.H., Kim, S.W., Park, H., Rahm, B., Pagilla, K. and Chandran, K. (2010) N2O Emissions from activated sludge Processes, 2008-2009: Results of a national monitoring survey in the United States. Environ Sci Technol 44(12), 4505–4511.

Bao, Z., Sun, S. and Sun, D. (2016) Assessment of greenhouse gas emission from A/O and SBR wastewater treatment plants in Beijing, China. International Biodeterioration & Biodegradation 108, 108-114.

Daelman, M.R., van Voorthuizen, E.M., van Dongen, U.G., Volcke, E.I. and van Loosdrecht, M.C. (2015) Seasonal and diurnal variability of N2O emissions from a full-scale municipal wastewater treatment plant. Sci Total Environ 536, 1-11.

Foley, J., de Haas, D., Yuan, Z. and Lant, P. (2010) Nitrous oxide generation in full-scale biological nutrient removal wastewater treatment plants. Water Res 44(3), 831-844.

Gujer, W. (2007) Siedlungswasserwirtschaft, Springer, Berlin.

Kimochi, Y., Inamori, Y., Mizuochi, M., Xu, K.-Q. and Matsumura, M. (1998) Nitrogen removal and N2O emission in a full-scale domestic wastewater treatment plant with intermittent aeration. Journal of Fermentation and Bioengineering 86(2), 202-206.

Masuda, S., Sano, I., Hojo, T., Li, Y.Y. and Nishimura, O. (2018) The comparison of greenhouse gas emissions in sewage treatment plants with different treatment processes. Chemosphere 193, 581-590.

Masuda, S., Suzuki, S., Sano, I., Li, Y.Y. and Nishimura, O. (2015) The seasonal variation of emission of greenhouse gases from a full-scale sewage treatment plant. Chemosphere 140, 167-173.

Mello, W.Z.d., Ribeiro, R.P., Brotto, A.C., Kligerman, D.C., Piccoli, A.d.S. and Oliveira, J.L.M. (2013) Nitrous oxide emissions from an intermittent aeration activated sludge system of an urban wastewater treatment plant. Química Nova 36, 16-20.

Ni, B.J., Pan, Y., van den Akker, B., Ye, L. and Yuan, Z. (2015) Full-Scale Modeling Explaining Large Spatial Variations of Nitrous Oxide Fluxes in a Step-Feed Plug-Flow Wastewater Treatment Reactor. Environ Sci Technol 49(15), 9176-9184.

Pan, Y., van den Akker, B., Ye, L., Ni, B.J., Watts, S., Reid, K. and Yuan, Z. (2016) Unravelling the spatial variation of nitrous oxide emissions from a step-feed plug-flow full scale wastewater treatment plant. Sci Rep 6, 20792.

Rodriguez-Caballero, A., Aymerich, I., Marques, R., Poch, M. and Pijuan, M. (2015) Minimizing N2O emissions and carbon footprint on a full-scale activated sludge sequencing batch reactor. Water Res 71, 1-10.

Rodriguez-Caballero, A., Aymerich, I., Poch, M. and Pijuan, M. (2014) Evaluation of process conditions triggering emissions of green-house gases from a biological wastewater treatment system. Sci Total Environ 493, 384-391.

Wang, Y., Fang, H., Zhou, D., Han, H. and Chen, J. (2016) Characterization of nitrous oxide and nitric oxide emissions from a full-scale biological aerated filter for secondary nitrification. Chemical Engineering Journal.
